# Combination therapy of vitamin C and thiamine for septic shock in a multicentre, double-blind, randomized, controlled study (ATESS): study protocol for a randomized controlled trial

**DOI:** 10.1186/s13063-019-3542-x

**Published:** 2019-07-11

**Authors:** Sung Yeon Hwang, Jong Eun Park, Ik Joon Jo, Seonwoo Kim, Sung Phil Chung, Taeyoung Kong, Jonghwan Shin, Hui Jai Lee, Kyoung Min You, You Hwan Jo, Doyun Kim, Gil Joon Suh, Taegyun Kim, Won Young Kim, Youn-Jung Kim, Seung Mok Ryoo, Sung-Hyuk Choi, Tae Gun Shin, for the Korean Shock Society (KoSS) Investigators

**Affiliations:** 1 Department of Emergency Medicine, Samsung Medical Centre, Sungkyunkwan University School of Medicine, 81 Irwon-ro, Gangnam-gu, Seoul, 06351 South Korea; 2 Statistics and Data Centre, Samsung Medical Centre, Seoul, South Korea; 3 Department of Emergency Medicine, Yonsei University College of Medicine, Seoul, South Korea; 4 Department of Emergency Medicine, Seoul National University College of Medicine, Seoul Metropolitan Government Seoul National University Boramae Medical Centre, Seoul, South Korea; 5 Department of Emergency Medicine, Seoul Metropolitan Government Seoul National University Boramae Medical Centre, Seoul, South Korea; 6 Department of Emergency Medicine, Seoul National University Bundang Hospital, Seongnam, South Korea; 7 Department of Emergency Medicine, Seoul National University College of Medicine, Seoul, South Korea; 8 Department of Emergency Medicine, Asan Medical Centre, University of Ulsan College of Medicine, Seoul, South Korea; 9 Department of Emergency Medicine, Guro Hospital, Korea University Medical Centre, Seoul, South Korea

**Keywords:** Sepsis, Septic shock, Thiamine, Vitamin C, Resuscitation

## Abstract

**Background:**

Septic shock is a life-threatening condition with underlying circulatory and cellular/metabolic abnormalities. Vitamin C and thiamine are potential candidates for adjunctive therapy; they are expected to improve outcomes based on recent experimental and clinical research. The aim of the Ascorbic Acid and Thiamine Effect in Septic Shock (ATESS) trial is to evaluate the effects of early combination therapy with intravenous vitamin C and thiamine on recovery from organ failure in patients with septic shock.

**Methods:**

This study is a randomized, double-blind, placebo-controlled, multicentre trial in adult patients with septic shock recruited from six emergency departments in South Korea. Patients will be randomly allocated into the treatment or control group (1:1 ratio), and we will recruit 116 septic shock patients (58 per group). For the treatment group, vitamin C (50 mg/kg) and thiamine (200 mg) will be mixed in 50 ml of 0.9% saline and administered intravenously every 12 h for a total of 48 h. For the placebo group, an identical volume of 0.9% saline will be administered in the same manner. The primary outcome is the delta Sequential Organ Failure Assessment (SOFA) score (ΔSOFA = initial SOFA at enrolment – follow-up SOFA after 72 h).

**Discussion:**

This trial will provide valuable evidence about the effectiveness of vitamin C and thiamine therapy for septic shock. If effective, this therapy might improve survival and become one of the main therapeutic adjuncts for patients with septic shock.

**Trial registration:**

ClinicalTrials.gov, NCT03756220. Registered on 5 December 2018.

**Electronic supplementary material:**

The online version of this article (10.1186/s13063-019-3542-x) contains supplementary material, which is available to authorized users.

## Background

Septic shock is a life-threatening disease; it is a subset of sepsis that involves organ dysfunction and profound underlying circulatory and cellular/metabolic abnormalities that cause death in more than 40% of patients [1, 2]. Not only does septic shock have high mortality, but the incidence rate is steadily rising due to the aging population and an increased rate of patients with comorbidities, making it very concerning from a public health perspective [3, 4].

Despite current standard treatments, including broad-spectrum antibiotics, source control, fluid resuscitation, and vasopressors, morbidity and mortality have remained high, and no adjunctive therapies for septic shock have been shown to improve survival [5, 6]. Moreover, although appropriate early resuscitation is applied to patients to improve their haemodynamic state, metabolic and oxidative stress can persist or even worsen at the cellular level, causing further organ dysfunction [1]. Additional interventions to improve patient-centred outcomes for septic shock thus need to be researched and developed [7].

Vitamin C and thiamine are soluble vitamins that are essential to human bodies. A deficiency can result in severe symptoms, including shock, cardiac failure, and central nervous system disorders [8]. Patients in septic shock show vitamin C and thiamine depletion, which can further aggravate septic shock [9, 10].

Vitamin C is a well-known antioxidant that is required as a cofactor/co-substrate for the biosynthesis of collagen, neurotransmitters, endogenous catecholamines, vasopressin, and cortisol [11]. Vitamin C enhances the synthesis of endogenous norepinephrine and vasopressin and increases adrenergic sensitivity [8, 12]. In animal and cell-based studies of sepsis, vitamin C acted as an antioxidant defence substance, reducing levels of reactive oxygen species and reactive nitrogen species and improving microcirculation by limiting oxidative injury and endothelial barrier disruption [13–17]. Vitamin C also has anti-inflammatory effects and plays a role in enhancing immune functions [8, 18].

Thiamine is a cofactor that acts on enzymes essential for glucose metabolism, the generation of adenosine triphosphate, and the production of nicotinamide adenine dinucleotide phosphate [19]. Thiamine deficiency causes bioenergetic failure, increases oxidative stress and cell injury, and accelerates organ failure, including brain dysfunction through neuronal injury [10, 20]. Thiamine administration to assist the metabolisms of patients in septic shock might be reasonable because of its acute consumption in the hypermetabolic state of septic shock and its important roles in cellular metabolism [21]. Thiamine might also reduce the risk of renal oxalate crystallization by preventing the conversion of vitamin C into oxalate, which is a potential adverse effect of vitamin C administration [8].

Recently, several preliminary studies about the clinical effects of vitamin C and thiamine have reported their results [22]. A phase I, randomized trial by Fowler et al. [23] included placebo (*n* = 8), low-dose intravenous vitamin C (50 mg/kg; *n* = 8), and high-dose vitamin C (200 mg/kg; *n* = 8); vitamin C reduced the levels of inflammatory markers, including C-reactive protein (CRP) and procalcitonin, in a dose-dependent manner in severe sepsis and septic shock. In another small randomized trial by Zabet et al. [24] involving 28 patients in the intensive care unit (ICU), the mean dose and duration of norepinephrine administration were significantly lower in the treatment group receiving intravenous vitamin C (100 mg/kg/day) than in the control group. Donnino et al. evaluated the effects of thiamine in a two-centre, randomized, double-blind trial in patients (*n* = 88) with septic shock. In the subgroup with thiamine deficiency (35%), those in the thiamine treatment group had lower lactate levels at 24 h and a decrease in mortality and the rate of renal replacement therapy (RRT) [25, 26]. Woolum et al. [27] used propensity matching in patients with septic shock and found that thiamine supplementation increased lactate clearance and decreased 28-day mortality. In a recently published before–after study (*n* = 94) by Marik et al. [28], combination therapy of vitamin C, thiamine, and steroid dramatically reduced mortality. A similar before–after study by Kim et al. [29] used the protocol from the study by Marik et al. and found a significant reduction in mortality among patients with severe pneumonia. However, these were both single-centre, retrospective observational studies, and debate remains about the effects of vitamin C and thiamine supplementation.

The theoretical and experimental evidence published to date suggests that vitamin C and thiamine supplementation can be used as an adjunctive therapy for septic shock [30]. Vitamin C and thiamine have the additional advantages of being inexpensive, widely available, and safe, with adverse effects occurring very rarely [31]. Until recently, however, their potential role has not been rigorously evaluated in a randomized clinical trial. In this trial of the potential advantages of vitamin C and thiamine, we hypothesize that their combined use will reduce organ failure in patients with septic shock.

### Objective

The Ascorbic Acid and Thiamine Effect in Septic Shock (ATESS) trial is designed to evaluate the effects of early combination therapy with intravenous vitamin C and thiamine on recovery from organ failure, as indicated by changes in the Sequential Organ Failure Assessment (SOFA) score during the first 72 h in patients with septic shock. We will also evaluate whether this therapy improves outcomes, including shock reversal and mortality, in patients with septic shock.

## Methods

### Study design

**Fig. 1:**
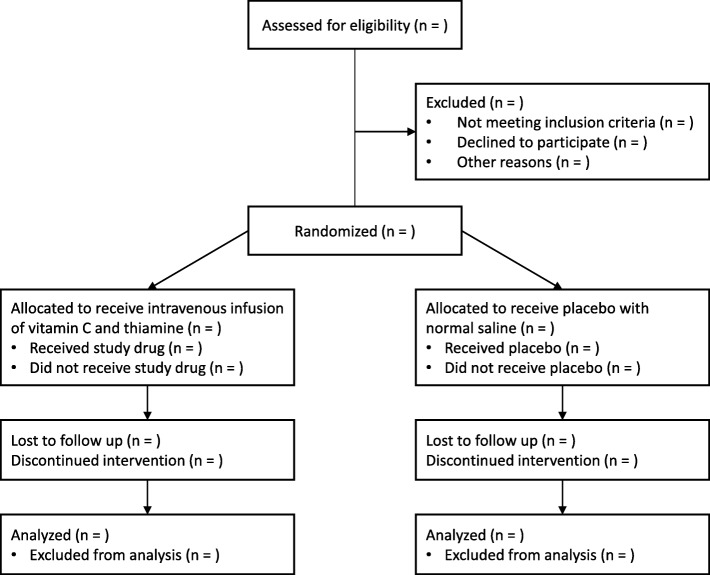
Study flow diagram

**Fig. 2:**
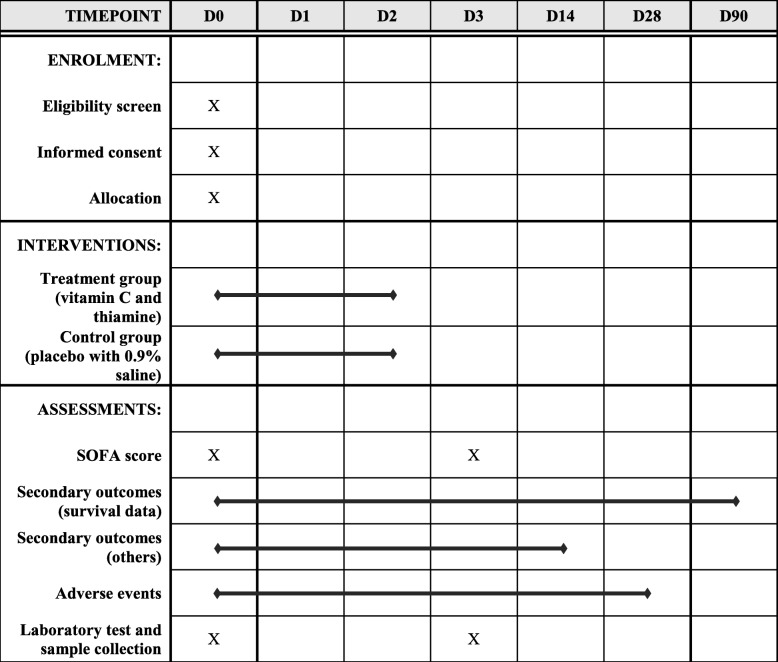
Schedule of enrolment, interventions, and assessments. SOFA Sequential Organ Failure Assessment

This study is a randomized, double-blind, placebo-controlled, multicentre trial in patients with septic shock. It will be conducted in six emergency departments (EDs) in South Korea. The trial protocol has been approved by the Institutional Review Boards of the individual participating hospitals and the Ministry of Food and Drug Safety in Korea. For all study participants, written informed consent will be obtained from either the patient or from the legal representative if the patient lacks capacity. Figures 1 and 2 show the study flow chart and the schedule, respectively. A Standard Protocl Items: Recommandations for Clinical Interventional Trials (SPIRIT) checklist is also available (Additional file 1).

### Eligibility criteria

Adult patients (19–89 years old) who visit an ED directly and are diagnosed with septic shock will be included. Septic shock is defined as sepsis with persisting hypotension requiring vasopressors to maintain a mean arterial pressure (MAP) ≥ 65 mmHg and with a serum lactate level > 2 mmol/L despite adequate volume resuscitation [2]. Sepsis is diagnosed in patients with suspected infection and organ dysfunction, which is identified as an acute change in the total SOFA score ≥ 2 points due to infection. If the baseline SOFA score is unknown, it will be assumed to be 0.

The following exclusion criteria apply: patients who are transferred from another hospital after vasopressor administration or mechanical ventilatory support; patients who have set limitations on treatment (e.g. patients with a signed do-not-resuscitate order); patients with an underlying terminal-stage disease without an active treatment plan and those who are not expected to survive to discharge; patients taking at least 1 g/day of vitamin C or receiving intravenous thiamine prior to enrolment; patients experiencing cardiac arrest prior to enrolment or who are expected to die within 24 h despite best possible treatment, based on the judgement of medical personnel; pregnant women; patients with glucose-6-phosphate dehydrogenase deficiency; patients with a history of hypersensitivity reactions to the trial drugs; patients with thalassemia; patients with hyperoxaluria; patients with cystinuria; patients with ongoing gout attacks; patients diagnosed with oxalate renal stones; patients who meet the inclusion criteria more than 24 h after ED arrival or who are screened more than 24 h after their diagnosis of septic shock; and patients who do not voluntarily consent to participate in the trial (directly or by legal proxy).

### Randomization and study medication

The patients will be randomly assigned in a 1:1 ratio to either the treatment group or the placebo group. The randomization sequence will be generated by an independent biostatistician using a permuted block size of four, stratified by recruitment site. The patients, treating clinicians, and researchers will be blinded to the allocated treatment throughout the trial. An identical number of ampoules of treatment drugs or placebo for each patient will be packed in advance. The trial packs, which contain the same number of ampoules of either vitamin C (500 mg/2 ml) and thiamine (50 mg/ 2 ml) or placebo (0.9% saline, 2 ml), were identically manufactured by Jeil Pharmaceutical (Daegu, South Korea). According to the order of allocation, the sequential randomization code for each site will be assigned to trial packs. When a patient in septic shock who meets the study criteria is identified, written consent will be obtained, and a unique randomization number will be assigned to each newly included participant in the order of enrolment. The treatment drug or placebo in the trial pack with the same number will be administered to the participant.

For the treatment group, vitamin C (50 mg/kg, maximum single dose 3 g, daily dose 6 g) and thiamine (200 mg) will be mixed in a 50-ml 0.9% saline bag and administered through intravenous infusion over 60 min every 12 h for a total of 48 h. For the placebo group, an identical volume of 0.9% saline from the placebo drug ampoule will be mixed in a 50-ml 0.9% saline bag and administered with the same protocol. Vitamin C and the placebo will be prepared in a sealed, opaque bag using a light-protective infusion set. The researcher may request unblinding of an independent pharmacist in the following situations: suspected severe adverse effects, including anaphylaxis, in which the researcher determines unblinding to be necessary for the benefit and safety of the patient; and when a patient or legal proxy requires an investigation about suspected damage caused by participation in the trial. Criteria for trial termination and dropout are as follows: if a patient or legal proxy requests termination of the trial or withdraws consent; if a patient is transferred to another hospital within 5 days of admission; if a patient or legal proxy does not follow the instructions of the physician in charge of the trial; if a patient develops a severe disease unrelated to participation in the trial; if the physician in charge of the trial determines that participation in the trial is not the best option for a given patient; and if a patient develops severe adverse effects due to the test drug including anaphylaxis.

### Clinical management

Initial resuscitation and management will be performed according to the latest Surviving Sepsis Campaign guidelines [32]. Broad-spectrum antibiotics will be administered as soon as possible, and another general treatment, such as source control, will be performed. Initially, 30 ml/kg of crystalloid fluids will be administered, but the dose may be titrated up or down depending on the patient’s condition. If the patient shows no response to fluids, norepinephrine will be administered to maintain a MAP of at least 65 mmHg. For patients requiring high-dose vasopressors (at least 0.2 μg/kg/min norepinephrine), additional vasopressin (0.03 U/min) and hydrocortisone (200 mg/day: 50 mg every 6 h or 200 mg over a 24-h continuous infusion) will be administered. Hydrocortisone will be administered until vasopressor therapy is stopped, for up to 7 days. Once a patient reaches and maintains the target MAP for at least 2 h, the dose of norepinephrine will first be reduced (0.1 μg/kg/min per hour) and then stopped completely, and the vasopressin dose will be reduced by 0.01 U per hour. This will be subject to modification based on the attending physician’s judgement and the patient’s status. Intravenous or enteral nutrient supply will be implemented, including the daily required amounts of vitamins to maintain normal nutrition. In cases in which vitamin C or thiamine deficiency is suspected and the attending physician judges it to be clinically necessary, open-label vitamin C (500 mg/day) or thiamine (100 mg/day) will be administered separately.

### Data and laboratory measurements

All data will be anonymized and collected using web-based report forms by investigators or trained research coordinators at each participating hospital who are blinded to the study group assignments. For quality control, data will be centrally reviewed at the coordinating hospital. Adverse events will be recorded both prospectively and by review of clinical charts (urticaria or rash, anaphylaxis, vomiting, diarrhoea, gastrointestinal bleeding, symptomatic nephrolithiasis, and others). Survival data will be collected from visiting patients, hospital records, and telephone interviews. Blood samples will be collected at enrolment prior to study drug administration and 72 h after enrolment for use in laboratory tests: complete blood count, creatinine, lactate, CRP, procalcitonin, vitamin C level, and thiamine level. We plan to collect and store serum samples at the same time. These samples will be stored for future biomarker analysis.

### Outcome measure

The primary outcome will be the ΔSOFA score, which is a 72-h change in SOFA score that reflects recovery from organ failure (ΔSOFA = initial SOFA at enrolment – follow-up SOFA after 72 h) [28, 33]. If a patient dies in the first 72 h, the previous worst score will be used. Secondary outcomes will be mortality (7-day, 28-day, 90-day, in-hospital, ICU death), time to death, time to shock reversal, vasopressor-free days, dose of vasopressor (at 24, 48, and 72 h from enrolment and maximum dose during 72 h), ventilator-free days, ventilator duration, RRT-free days, new use of RRT, new onset or aggravation of acute kidney injury (AKI), length of ICU stay, ICU-free days, length of hospital stay, CRP change during the initial 72 h (%), and procalcitonin change during the initial 72 h (%). For CRP and procalcitonin, if a patient dies within the first 72 h, the last follow-up values will be used. Shock reversal is defined as discontinuation of all vasopressors and MAP maintained at 60 mmHg or more for longer than 24 h [34]. For outcome variables showing free days, the time frame will be from enrolment day to day 14. If a patient dies before day 14, the free days after death will be considered 0. The dose of vasopressor is expressed as a norepinephrine equivalent dose [35]. AKI is defined as Kidney Disease: Improving Global Outcomes (KDIGO) stage 2 or higher, which is new-onset change [36]. For evaluation of the KDIGO stage, baseline creatinine is derived from the lowest value from 1 year to 24 h prior or estimated by a predefined formula (creatinine = 0.74–0.2 (if female) + 0.003 × age) if the former is unavailable [37]. Patients receiving dialysis for chronic end-stage renal failure will be excluded from the analysis.

### Statistical analysis

The sample size was calculated based on the ΔSOFA score. With reference to the ΔSOFA scores of a control group in a study by Marik et al. [28], we anticipate the mean and standard deviations of the ΔSOFA scores in the control group to be 1.0 and 2.7, respectively, and the mean ΔSOFA score in the test group to be at least 2.5. This effect size of a 1.5-point improvement in the 72-h SOFA score is set as a surrogate value for a survival benefit in patients. Assuming that the standard deviation of the ΔSOFA score is the same in both groups, with a statistical significance of 5% and power of 80%, the required participant number per group is 52 patients. Assuming a dropout rate of 10%, we determined that 58 patients will be required in each group (total *n* = 116).

Data analyses will be conducted with the intention-to-treat set, the full analysis set, and the per-protocol analysis set, and missing data will not be imputed. For the primary outcome analysis, the ΔSOFA scores will be compared using *t* tests or the Mann–Whitney test, depending on the distribution between the two groups. For secondary outcomes and adverse events, we will use *t* tests or the Mann–Whitney test for continuous variables and the chi-square test or Fisher’s exact test for categorical variables. Linear mixed models and generalized linear mixed models will be used for multivariable analyses of continuous outcomes and categorical outcomes, respectively, adjusting for study centre (random effect) and the covariates (fixed effect) of age, sex, infection foci, underlying disease, SOFA score, initial lactase level, and albumin level, if the covariates are unbalanced between the test group and the control group. Survival duration and time to shock reversal will be analysed using the Kaplan–Meier method and compared by the log-rank test. Subgroup analysis will be conducted by predefined groups according to age, sex, infection site, norepinephrine dose at randomization, SOFA score, hypoalbuminemia, adjunctive steroid use, time from hypotension to trial drug administration, malignancy, vitamin C level, and thiamine level. *P* < 0.05 will be considered statistically significant and corrected using Bonferroni’s method for multiple testing. Statistical analysis will be performed using SAS version 9.4 (SAS Institute, Cary, NC, USA) and STATA version 15.1 (STATA Corporation, College Station, TX, USA). A data monitoring committee will be established to review trial data upon recruiting half of the planned patients. This will consist of an independent physician who is experienced with clinical trial implementation and a biostatistician. The committee will evaluate differences in the primary outcome to decide whether the researcher terminates the trial or continues, and will assess adverse events.

## Discussion

Treatment of septic shock is challenging, and mortality is high despite maximal intensive care [6]. Moreover, most clinical trial interventions for septic shock have failed to improve outcomes, so the development of effective treatments is important [7]. Vitamin C and thiamine are potential candidates for adjunctive therapy for septic shock. These are safe treatments expected to improve prognosis based on recent experimental and clinical studies [8]. The key goal of this study is to assess the effects of early combination therapy with intravenous vitamin C and thiamine on recovery from organ failure in patients with septic shock. This trial, along with current ongoing trials [30], will provide important evidence about the effectiveness of vitamin C and thiamine supplementation for septic shock when administered early in the ED. If this therapy is shown to be effective, this regimen would be immediately applicable to the management of septic shock and could become one of the main therapeutic adjuncts.

In this trial, we have chosen the ΔSOFA score as the primary endpoint. A relatively large number of patients would be required to evaluate a survival difference. The ΔSOFA score is well correlated with survival and reflects recovery from organ failure [33, 38, 39]. Therefore, this primary outcome measurement might be a reasonable approach to complement the limitation of a relatively small sample size. In addition, we will measure several important secondary endpoints, including mortality and shock reversal time.

Steroid use can reduce the duration of vasopressor use and enhance recovery from shock, although there is no definite evidence that it improves survival [34]. Steroids can be used with vitamin C for synergistic effects, and other trials have proposed this combination [28, 30, 40]. In this trial, we will add steroid treatment only for patients requiring a high dose of vasopressor, in accordance with the current Surviving Sepsis Campaign guidelines [32]. We will perform subgroup analyses according to concomitant use of a steroid, which might provide data about potential treatment synergism.

Regarding vitamin C and thiamine use for septic shock patients, the pharmacokinetics, optimal dosage, and duration need to be investigated [22]. In addition to the clinical outcomes, this trial will allow us to evaluate the effects of vitamin C and thiamine supplementation during the initial 48-h period in septic shock, which has higher metabolic demands, on blood levels of vitamin C and thiamine.

## Trial status

Currently recruiting. Trial start date was 1 December 2018. Anticipated recruitment end date is December 2019.

## Additional file

Additional file 1: SPIRIT 2013 checklist: recommended items to address in a clinical trial protocol and related documents. (DOC 125 kb)

## Data Availability

The datasets generated and/or analysed during the study will be available from the corresponding author on reasonable request. The findings of this study will be shared through medical literatures and conferences.

## Abbreviations

AKI: Acute kidney injury
ATESS: Ascorbic Acid and Thiamine Effect in Septic Shock
CRP: C-reactive protein
ED: Emergency department
ICU: Intensive care unit
KDIGO: Kidney Disease: Improving Global Outcomes
MAP: Mean arterial pressure
RRT: Renal replacement therapy
SOFA: Sequential Organ Failure Assessment
